# Vision-related quality of life after surgery for vitreoretinal disorders in a Mexican population: an observational study

**DOI:** 10.1038/s41598-023-32152-z

**Published:** 2023-03-25

**Authors:** Ilse Sarahí Márquez-Vergara, Geovanni Jassiel Ríos-Nequis, Ingrid Yazmín Pita-Ortíz, Héctor Javier Pérez-Cano, Selma Alin Somilleda-Ventura

**Affiliations:** 1 Ophthalmology Service, Fundación Hospital Nuestra Señora de la Luz IAP, Mexico City, Mexico; 2 Retina and Vitreous Department, Fundación Hospital Nuestra Señora de la Luz IAP, Mexico City, Mexico; 3 Biomedical Research Center, Fundación Hospital Nuestra Señora de la Luz IAP, Ezequiel Montes 135, Tabacalera, Cuauhtémoc, 06030 Mexico City, Mexico; 4 Interdisciplinary Center of Health Sciences, National Polytechnic Institute, Mexico City, Mexico

**Keywords:** Psychology, Diseases, Medical research

## Abstract

Visual-related quality of life in retinal diseases has not been explored in the Mexican population, so the study aims to identify it in patients undergoing surgery due to advanced diabetic retinopathy, rhegmatogenous retinal detachment, and other causes of vitrectomy; the Visual Function Quality-25 questionnaire was applied to 76 patients, pre-and postoperative. It was divided into 10 domains and interpreted according to the National Eye Institute scores, where the highest value was the best visual function. Student's t-test for related samples and Wilcoxon’s t-test were used to compare each domain between measurements, and Pearson’s R test to correlate the total score of age and quality of life; a *p* value < 0.05 was considered significant. Diabetic retinopathy patients showed an improvement 1 and 3 months after surgery in all domains; in rhegmatogenous retinal detachment, there was an improvement observed up to 3 months, while a decrease in ocular pain was observed in other causes of vitrectomy. Differences found in all the quality-of-life scores were not statistical, but clinically significant. The study shows that visual-related quality of life domains improves after vitrectomy; the inclusion of this analysis might be considered relevant within the parameters of surgical success of the most prevalent vitreoretinal diseases.

## Introduction

The World Health Organization (WHO) defines quality of life as the perception that an individual has in the context of their culture, the values in which they live, their expectations, norms, and concerns^1^. Consists of both objective components and material circumstances of life, for example, illness, pain, disability, and other factors added to subjective components that are mainly the degree of satisfaction and the perception of living conditions^2^.

Currently, it is not enough to assess the value of visual acuity and the visual field as surgical success, but the quality of life perceived after eye surgery must be included. There are some instruments to assess the quality of life and visual function, including The Visual Disability Assessment (VF-14)^3^, the Activities of Daily Vision Scale (AVDS)^4^, the Quality of Life Questionnaire (QOLQ)^5^, Visual Function Quality-39 (VFQ-39)^6^ and Visual Function Quality-25 (VFQ-25)^7^; the latter has demonstrated validity and reliability for different populations in distinct languages in the 25-item adaptation derived from the original 51-item^8^.

The questionnaire measures the influence of visual disability and visual symptoms in physical and mental health domains and those related to visual functioning, problems involving vision, or the patient’s perception of their vision condition^9^. It has been evaluated in Spanish^10^, for Latino patients living in the United States^9^, Cubans^8^, and Colombians^11^, where internal reliability coefficients are lower than those necessary in the items of general health and driving (so they are eliminated from the questionnaire in its Spanish version)^8,10^.

It has been translated and adapted to the cultural environment and validated in Serbian, Turkish, Chinese, Japanese, Greek, French, Italian, Polish, Portuguese, and Spanish^9,10^. Among the retinal pathologies in which it has been evaluated, Okamoto and his group^12^ report a higher preoperative and postoperative score for macular hole and epiretinal membrane and retinal detachment. The VQF-25 has become a tool that has been used to assess the quality of life in subjects with cataracts^13^, glaucoma^14^, age-related macular degeneration^15^, and diabetic retinopathy^16^, among others.

However, the VFQ-25 questionnaire in its Spanish version has not been applied to the most prevalent retinal pathologies in Mexico. Our objective was to identify the quality-of-life score related to vision in patients with vitreoretinal diseases who undergo retinal surgery in a reference ophthalmological hospital and determine which are the domains of quality of life with higher impact after surgery.

## Methods

It is an observational, prospective, comparative, and longitudinal study. Seventy-six patients between 18 and 80 years, who had diabetic retinopathy, rhegmatogenous retinal detachment, and other vitreoretinal pathologies (macular hole, epiretinal membrane, and causes of vitreous hemorrhage) in one eye, were included by non-probabilistic sampling determined by time; patients with other pathologies such as cataracts or glaucoma that compromises the visual function were excluded. Patients were followed-up after a vitrectomy at 1 and 3 months in person or by telephone between May and August 2019; they agreed to participate, understood, and signed the informed consent. This study followed the principles established in the Declaration of Helsinki and was approved by the Institutional Review Board of the hospital where it took place. The data was handled and analyzed with strict confidentiality and according to good practices.

The demographic variables considered were age and sex, and the best-corrected visual acuity (BCVA) was recorded before the intervention, which was converted to the minimum angle of resolution (logMAR) for statistical calculations. Within the approximations, finger count was considered as 20/2000, hand movement = 20/4000, and light perception = 20/8000.

The researchers applied the Visual Function Quality-25 test; the questionnaire was divided into 10 domains: (1) general vision, (2) eye pain, (3) close activities, (4) distance activities, (5) social functioning, (6) mental health, (7) role limitations, (8) dependence, (9) color vision, and (10) peripheral vision. The Spanish version excludes the general health and driving items from the algorithm, for which there are 23 items^8^; its application time was less than 15 min. The researchers explained the questionnaire to the patients at the beginning of the application and provided additional support when necessary.

For the calculation and interpretation of the results, the National Eye Institute (NEI) scoring algorithm was used^17^, which considers the subscales with a score from 0 to 100, where 100 is the highest function. The total score was the mean of all items; in case an answer to a question was not related to vision or did not apply to the context, it was excluded from the overall score.

The Kolmogorov–Smirnov statistical test evaluated the data distribution; only items 3, 7, and 8 in the diabetic retinopathy group did not have a normal distribution. The means of each domain with missing data imputed by the last observation carried forward (LOCF) were compared using Student’s t-test for related samples and Wilcoxon’s t-test, as appropriate, and the variable quality of life total score between the different types of tamponades was calculated using Kruskal–Wallis. Also, the correlation coefficient of the total score concerning age was obtained by Pearson’s R test, which considered an r < 0.4 as a weak correlation, r ≥ 0.4–0.7 as moderate, and r > 0.7 as a strong correlation; a *p* value < 0.05 was considered significant. Data were stored and analyzed in GraphPad Prism version 8.0 for Mac.

### Ethics approval

Approval was obtained from the ethics committee of Fundación Hospital Nuestra Señora de la Luz IAP. The procedures used in this study adhere to the tenets of the Declaration of Helsinki.

### Consent to participate

Informed consent was obtained from all individual participants included in the study.

## Results

Of the presurgical sample, the mean age was 50.4 ± standard deviation (S.D.) 10.19 years, 44 men (57.89%) and 32 women (42.11%); 54% of the patients undergoing surgery for advanced diabetic retinopathy (n = 41), 30% for rhegmatogenous retinal detachment (n = 23), and 12% for other causes of vitrectomy (n = 12).

Forty-one patients diagnosed with advanced diabetic retinopathy aged 53.27 years ± 9.06; 61% (n = 25) were men. For this group, the preoperative quality-of-life total score was 43.01 ± 18.19 points, after 1 month 57.62 ± 20.95, and at 3 months 59.17 ± 19.11 (Table 1); the rest of the items also showed improvement in quality of life at 1 month, although the subjects evaluated reported higher ocular pain after surgery than before it (*p* < 0.001). This statistical difference persisted when the pre-surgical evaluation was compared with the 3 months follow-up after the intervention, except in the general vision and social performance items, although in both cases quality of life improved.

**Table 1:** Comparison of quality-of-life related variables of pre-and post-surgical evaluations, in advanced diabetic retinopathy.

Domain	PreOp versus 1-month*		PreOp versus 3-months*		One month versus 3 months*	
	Mean (SD)	*p*	Mean (SD)	*p*	Mean (SD)	*p*
Total score	43.00 (18.18)–57.51 (20.94)	< 0.001	43.00 (18.18)–59.17 (19.11)	0.001	57.51 (20.94)–59.17 (19.11)	0.60
General vision	41.95 (14.70)–65.52 (23.23)	0.03	41.95 (14.70)–63.95 (18.15)	0.41	65.52 (23.23)–63.95 (18.15)	0.16
Ocular pain	85.67 (15.94)–73.27 (23.08)	< 0.001	85.67 (15.94)–81.57 (17.86)	< 0.001	73.27 (23.08)–81.57 (17.86)	0.19
Near vision**	16.67 (8.33–45.83)–50 (25–79.17)	< 0.001	16.67 (8.33–45.83)–50 (33.33–75)	0.001	50 (25–79.17)–50 (33.33–75)	0.20
Distance vision	39.32 (27.99)–52.68 (29.24)	< 0.001	39.32 (27.99)–51.09 (29.32)	0.001	52.68 (29.24)–51.09 (29.32)	0.91
Social functionality	43.90 (32.37)–64.65 (31.53)	0.04	43.90 (32.37)–63.81 (29.43)	0.12	64.65 (31.53)–63.81 (29.43)	0.94
Mental health	32.93 (26.33)–44.18 (26.51)	0.008	32.93 (26.33)–43.42 (29.71)	0.01	44.18 (26.51)–43.42 (29.71)	0.29
Role limitations**	25 (0–50)–50 (25–75)	0.02	25 (0–50)–50 (25–75)	0.01	50 (25–75)–50 (25–75)	0.30
Dependence**	25 (0–50)–50 (16.67–83.33)	0.002	25 (0–50)–50 (25–75)	< 0.001	50 (16.67–83.33)–50 (25–75)	0.74
Color vision	75 (34.46)–81.89 (26.64)	0.001	75 (34.46)–77.63 (23.41)	< 0.001	81.89 (26.64)–77.63 (23.41)	0.73
Peripheral vision	46.34 (22.05)–65.52 (29.43)	< 0.001	46.34 (22.05)–69.74 (24.41)	< 0.001	65.52 (29.43)–69.74 (24.41)	0.74

*PreOp* Preoperative.

*Paired student’s t-test.

**Wilcoxon’s t test.

**Figure 1:**
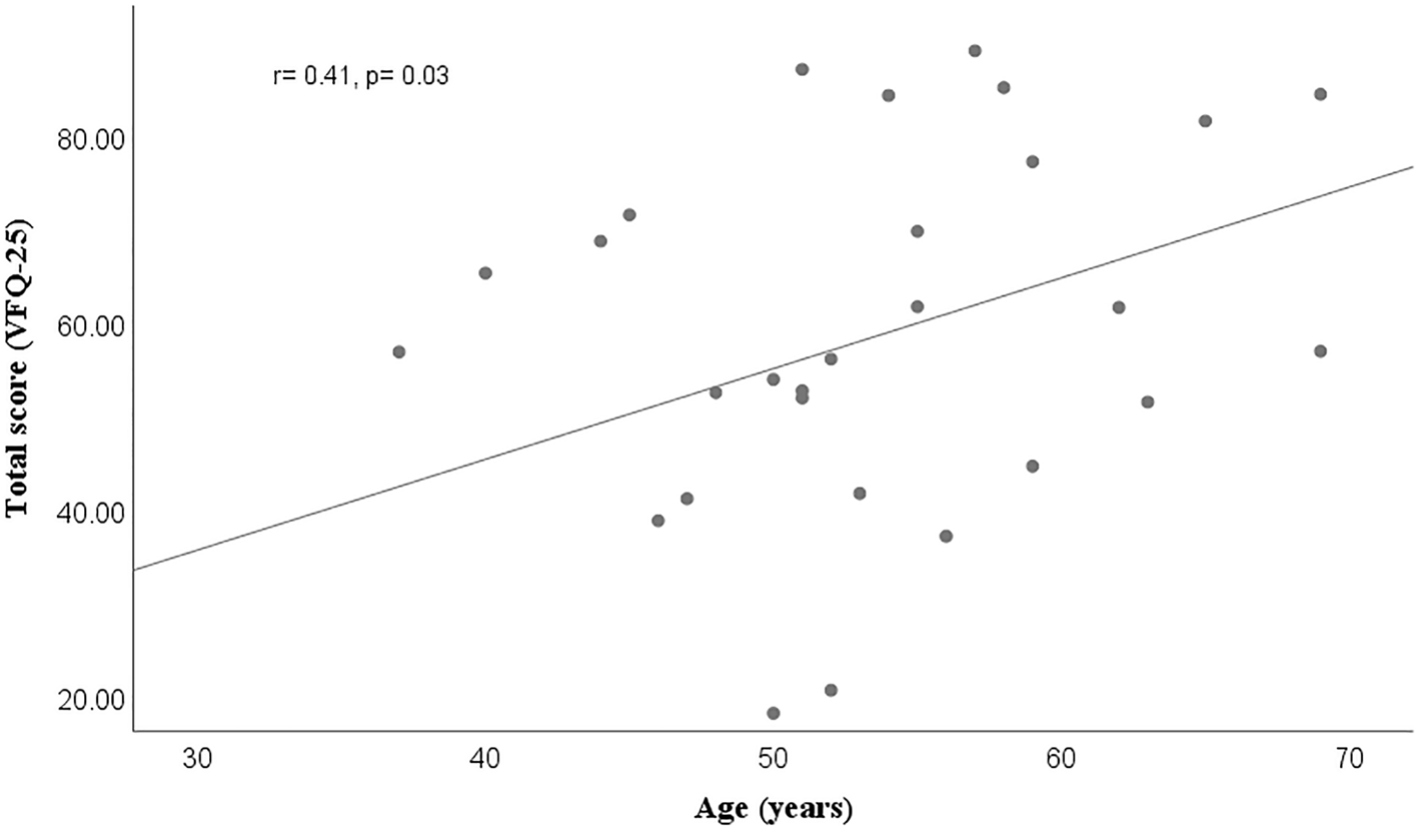
Moderate positive linear correlation between age and quality-of-life total score in diabetic retinopathy subjects, 1 month after undergoing vitrectomy.

**Figure 2:**
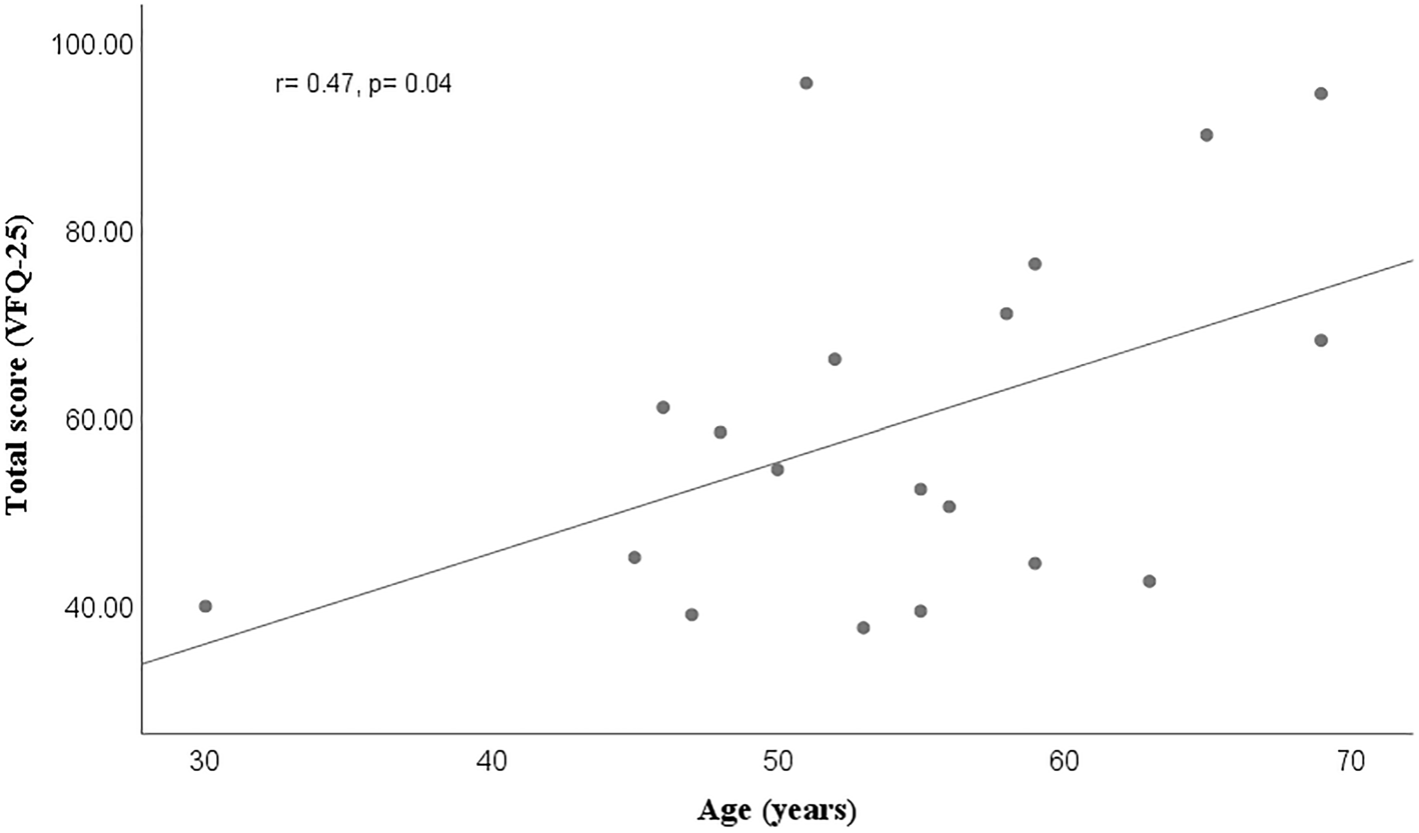
Moderate positive linear correlation between age and quality-of-life total score in diabetic retinopathy subjects, 3 months after undergoing vitrectomy.

Neither of the cases had a significant difference in the scores obtained when the 1-month and 3 months after surgery; there was also no difference between sex and the quality-of-life total score before surgery (*p* = 0.27), at 1 month (*p* = 0.93), and 3 months (*p* = 0.18). Regarding the type of tamponade, there was a higher quality of life pre-surgical score when the tamponade was air/gas versus silicone (*p* = 0.04). The correlation between age and the pre-surgical total score had a weak positive linear correlation (r = 0.15, *p* = 0.34), but this changed when evaluating the total score 1 month (r = 0.43) and 3 months (r = 0.47) after surgery, where existed moderate positive correlations with statistical significance (*p* < 0.05, Figs. 1, 2).

In patients undergoing surgery for rhegmatogenous retinal detachment, the mean age was 51.43 ± 12.31 years; 61% (n = 14) were men. The evaluation included 15 right eyes and eight left eyes. The higher quality of life scores were mental health and role difficulty items 1 month after surgery compared to the pre-surgical evaluation (*p* < 0.05, Table 2); however, the difference did not appear between the two post-surgical total scores. Five items showed significant improvement 3 months after surgery, and only the ocular pain variable had a higher score before surgery. There was also no change at 1 month and 3 months after surgery in the pre-surgical total score concerning patient sex or the type of tamponade (*p* > 0.05). In the correlation analysis, existed negative linear relations between age and the total score at 1 month (r = -0.44) and 3 months (r = -0.27), although there was no statistical difference (*p* > 0.05).

**Table 2:** Comparison of quality-of-life related variables of pre-and post-surgical evaluations, in rhegmatogenous retinal detachment.

Domain	PreOp versus 1-month*		PreOp versus 3-months*		One month versus 3 months*	
	Mean (SD)	*p*	Mean (SD)	*p*	Mean (SD)	*p*
Total score	58.04 (18.30)–61.65 (17.77)	0.02	58.04 (18.30)–67.88 (11.01)	0.06	61.65 (17.77)–67.88 (11.01)	0.99
General vision	46.09 (12.70)–66.67 (10)	0.09	46.09 (12.70)–65.71 (9.76)	0.04	66.67 (10)–65.71 (9.76)	0.99
Ocular pain	85.76 (20.54)–79.16 (20.73)	0.59	85.76 (20.54)–82.14 (14.17)	0.04	79.16 (20.73)–82.14 (14.17)	0.21
Near vision	48.19 (28.42)–49.07 (33.18)	0.11	48.19 (28.42)–65.48 (12.20)	0.19	49.07 (33.18)–65.48 (12.20)	0.74
Distance vision	59.78 (34.23)–62.96 (34.45)	0.64	59.78 (34.23)	0.15	62.96 (34.45)–	0.39
Social functionality	67.93 (33.03)–63.89 (36.14)	0.35	67.93 (33.03)–65.48 (27.92)	0.18	63.89 (36.14)–65.48 (27.92)	0.39
Mental health	44.84 (25.95)–51.39 (220.5)	0.02	44.84 (25.95)–76.78 (34.93)	0.002	51.39 (220.5)–76.78 (34.93)	0.69
Role limitations	25 (0–50)–62.50 (12.50–87.50)	0.02	25 (0–50)–75 (37.50–87.50)	0.02	62.50 (12.50–87.50)–75 (37.50–87.50)	0.71
Dependence	57.61 (35.35)–65.74 (29.30)	0.99	57.61 (35.35)–70.23 (23)	0.41	65.74 (29.30)–70.23 (23)	0.36
Color vision	90.21 (19.57)–83.33 (21.65)	0.24	90.21 (19.57)–89.28 (13.36)	0.03	83.33 (21.65)–89.28 (13.36)	0.69
Peripheral vision	58.69 (22.12)–75 (33.07)	0.14	58.69 (22.12)–78.57 (22.49)	0.02	75 (33.07)–78.57 (22.49)	0.52

*PreOp* Preoperative.

*Paired student’s t-test.

Regarding other causes of vitrectomy analyzed, the mean age was 53.17 ± 10.12 years; 58.3% (n = 7) were women. The evaluation included 11 right eyes and one left. The quality-of-life total score was 56.14 ± 10.11 before surgery, 66.49 ± 18.63 at 1 month, and 63.59 ± 24.59 at 3 months. Only the ocular pain variable showed a higher quality of life after surgery with statistical significance when compared with pre-surgical evaluation (Table 3). Quality-of-life items compared with the type of tamponade or patient sex had no difference but a weak positive correlation between age and the pre-surgical total score, not significant (r = 0.17, *p* = 0.59).

**Table 3:** Comparison of quality-of-life related variables of pre-and post-surgical evaluations, in other causes of vitrectomy.

Domain	PreOp versus 1-month*		PreOp versus 3-months*		One month versus 3 months*	
	Mean (SD)	*p*	Mean (SD)	*p*	Mean (SD)	*p*
Total score	56.14 (14.44)–66.49 (18.62)	0.27	56.14 (14.44)–63.59 (24.32)	0.44	66.49 (18.62)–63.59 (24.32)	0.73
General vision	56.67 (14.35)–54 (12.65)	0.17	56.67 (14.35)–64.44 (19.43)	0.40	54 (12.65)–64.44 (19.43)	0.91
Ocular pain	81.25 (17.27)–71.25 (24.33)	0.002	81.25 (17.27)–66.67 (31.87)	0.008	71.25 (24.33)–66.67 (31.87)	0.67
Near vision	33.33 (21.90)–58.33 (27.78)	0.87	33.33 (21.90)–62.03 (25.72)	0.61	58.33 (27.78)–62.03 (25.72)	0.50
Distance vision	61.11 (19.08)–65.41 (28.87)	0.18	61.11 (19.08)–59.72 (28.87)	0.99	65.41 (28.87)–59.72 (28.87)	0.17
Social functionality	63.54 (22.27)–77.50 (22.67)	0.14	63.54 (22.27)–66.67 (32.47)	0.87	77.50 (22.67)–66.67 (32.47)	0.14
Mental health	48.44 (23.10)–61.87 (18.97)	0.53	48.44 (23.10)–51.39 (23.13)	0.33	61.87 (18.97)–51.39 (23.13)	0.18
Role limitations	41.66 (35.89)–50 (28.87)	0.27	41.66 (35.89)–58.33 (27.95)	0.61	50 (28.87)–58.33 (27.95)	0.90
Dependence	54.86 (28.30)–69.17 (30.44)	0.68	54.86 (28.30)–68.52 (34.80)	0.22	69.17 (30.44)–68.52 (34.80)	0.17
Color vision	93.75 (15.54)–90 (21.08)	0.05	93.75 (15.54)–83.33 (27.95)	0.59	90 (21.08)–83.33 (27.95)	0.41
Peripheral vision	54.17 (20.87)–72.22 (23.20)	0.14	54.17 (20.87)–63.89 (35.60)	0.58	72.22 (23.20)–63.89 (35.60)	0.77

*PreOp* Preoperative.

*Paired student’s t-test.

## Discussion

Our study found an increase in quality of life 1 month after vitrectomy in each group and increased up to 16 points when compared to pre-surgical vision quality versus 3 months after vitrectomy evaluation, in patients with advanced diabetic retinopathy, 9 points in patients with rhegmatogenous retinal detachment, and 6 points in patients undergoing surgery for other reasons. The type of air/gas tamponade showed a better quality of life score compared to those in which silicone was used, this may be because its use is generally limited to the most complex retinal disease cases who perceive a lower quality of life-related to vision; additionally, a correlation was identified between older age and an improvement in the quality-of-life score after surgery, particularly in patients with diabetic retinopathy.

These findings contrast with the reported by Okamoto and his group^18^, who applied the test to 51 patients diagnosed with diabetic retinopathy before undergoing surgery and 3 months later; the score reported in vitreous hemorrhage was 51.2 to 62.3, tractional retinal detachment 61.1 to 70.3 and excessive macular traction 55.2 to 59.4. The subscales that reported significant improvement after surgery were general vision, near and distance activities, social function, mental health, role difficulty, driving, and peripheral vision. However, no correlation was shown with age, duration of diabetes, HbA1C levels, or fasting glucose. Our study observed that the preoperative score was below the reported by Okamoto (43.00); however, the results after surgery are similar (57.51 and 59.17), indicating a significant improvement in the quality of life of patients undergoing vitrectomy in our country.

The quality-of-life total score in cases of rhegmatogenous retinal detachment has been reported at 80 points in women and 74.7 in men with follow-up at 6 months; according to Smeretschnig, the items with the greatest difference concerning normal controls were general vision, mental health, social functionality, driving, and color vision^20^. On the other hand, a group reported this difference at 3 months of operated patients versus controls, in near activities, peripheral vision, dependency, and mental health^19^, whose findings coincide with those of Du and his team^21^, although the latter applied the questionnaire one day before surgery. We compared the same patients over time and found a significant difference in the composite score, regarding mental health and role difficulty during the first month, while at 3 months these same variables are preserved, general vision showed an improvement, and eye pain, color vision, and peripheral vision scores decreased; it may be possible that the changes reported in each item are higher the more time passes after surgery, but this requires an additional long-term study.

In epiretinal membranes, vitrectomy has significantly improved the components of quality of life, being the most important post-surgical improvement among the pathologies studied^12^. It mainly impacts 10 subscales except for peripheral vision and general health (mean score 77.9). The results seem to be directly related to the presence and severity of preoperative metamorphopsia, but not visual acuity, contrast sensitivity, or central macular thickness^22^. On the contrary, Ghazi reports that there is an improvement of the metamorphopsia perception after surgery, and considers that the initial visual acuity correlates with the initial VFQ-25 score; however, in his study, VA does not improve considerably after surgery, and only remote activities, general vision, and the overall score had an improvement^23^. Matsuoka^24^ has reported that the greatest improvement-related items at 3 months after surgery are visual capacity, general vision, close activities, role difficulties, and the composite score, while at 12 months it is the improvement of the metamorphopsia-perception, general vision, close activities, distance activities, mental health, role difficulty, and the composite score. In this study, we found no difference in the pre-and post-surgical quality of life for this pathology, which was included in other causes of vitrectomy; the only item with a statistical difference was the eye pain that improved after surgery in the first month. However, further analysis is required to evaluate the quality of life and its correlation with the visual function test.

It is important to consider the quality of life as one of the variables within surgical success that are observed to increase, after surgery for retinal pathologies, which is more noticeable in older patients (43% in the evolution at 1 month), and in 47% of the cases 3 months after the intervention, which represents a 30% of change concerning the quality of life prior the vitrectomy, according to our findings. The relevance lies in the high prevalence of these diseases in our population and their poor control; to our knowledge, it is the first report of quality of life-related to vision in patients undergoing vitrectomy in Mexico. However, we recognized that it is necessary to generate effective strategies for achieving a complete follow-up in all temporalities of the evaluation, to limit a possible bias along the analysis; this must be taken into account for further studies.

In conclusion, vitrectomy performed in patients with advanced diabetic retinopathy improves the quality-of-life score associated with vision and in each of the items from the first month after surgery, while for rhegmatogenous retinal detachment, the relevant improvement is observed up to 3 months after surgery. Another prospective study with a long-term follow-up that considers vision and sensory tests is suggested, in addition to increasing the sample of patients.

## Data Availability

The datasets generated during and/or analyzed during the current study are available from the corresponding author upon reasonable request.
